# The Red Harmful Plague in Times of Climate Change: Blooms of the Cyanobacterium *Planktothrix rubescens* Triggered by Stratification Dynamics and Irradiance

**DOI:** 10.3389/fmicb.2021.705914

**Published:** 2021-08-25

**Authors:** Deborah Knapp, Bieito Fernández Castro, Daniel Marty, Eugen Loher, Oliver Köster, Alfred Wüest, Thomas Posch

**Affiliations:** ^1^Limnological Station, Department of Plant and Microbial Biology, University of Zurich, Kilchberg, Switzerland; ^2^Ocean and Earth Science, National Oceanography Centre, University of Southampton, Southampton, United Kingdom; ^3^Zurich Water Supply, Zurich, Switzerland; ^4^Physics of Aquatic Systems Laboratory, Margaretha Kamprad Chair, Institute of Environmental Engineering, École Polytechnique Fédérale de Lausanne, Lausanne, Switzerland; ^5^Eawag, Swiss Federal Institute of Aquatic Science and Technology, Surface Waters – Research and Management, Kastanienbaum, Switzerland

**Keywords:** cyanobacteria, cyanoHAB, lake warming, long-term data, metalimnetic species, neutral buoyancy, deep convective mixing, deep chlorophyll maximum

## Abstract

*Planktothrix rubescens* is a harmful planktonic cyanobacterium, forming concentrated metalimnetic populations in deep oligo- and mesotrophic lakes, even after successful restoration. In Lake Zurich (Switzerland), *P. rubescens* emerged as a keystone species with annual mass developments since the 1970s. Its success was partly attributed to effects of lake warming, such as changes in thermal stratification and seasonal deep mixing. However, recent observations based on a biweekly monitoring campaign (2009–2020) revealed two massive breakdowns and striking seasonal oscillations of the population. Here, we disentangle positive from negative consequences of secular lake warming and annual variations in weather conditions on *P. rubescens* dynamics: (i) despite the high survival rates of overwintering populations (up to 25%) during three consecutive winters (2014–2016) of incomplete deep convective mixing, cyanobacterial regrowth during the following stratified season was moderate and not overshooting a distinct standing stock threshold. Moreover, we recorded a negative trend for annual population maxima and total population size, pointing to a potential nutrient limitation after a series of incomplete winter mixing. Thus, the predication of steadily increasing blooms of *P. rubescens* could not be confirmed for the last decade. (ii) The seasonal reestablishment of *P. rubescens* was strongly coupled with a timely formation of a stable metalimnion structure, where the first positive net growth in the following productive summer season was observed. The trigger for the vertical positioning of filaments within the metalimnion was irradiance and not maximal water column stability. Repetitive disruptions of the vernal metalimnion owing to unstable weather conditions, as in spring 2019, went in parallel with a massive breakdown of the standing stock and marginal regrowth during thermal stratification. (iii) Driven by light intensity, *P. rubescens* was entrained into the turbulent epilimnion in autumn, followed by a second peak in population growth. Thus, the typical bimodal growth pattern was still intact during the last decade. Our long-term study highlights the finely tuned interplay between climate-induced changes and variability of thermal stratification dynamics and physiological traits of *P. rubescens*, determining its survival in a mesotrophic temperate lake.

## Introduction

Large lakes in Central Europe are in many cases the zones of densely populated settlements and provide essential local ecosystem services. However, temperate lakes are susceptible to climate change (Salmaso et al., 2018), especially to the multidecadal increase of air temperature and solar radiation (Schmid and Köster, 2016). The warming of surface water strata is comprehensively documented (O’Reilly et al., 2015), especially for spring and summer periods (Yankova et al., 2016), as well as the reduced cooling during winter (Dokulil, 2013). These changes in the heat balance are reflected by stronger thermal stratification of the water column which results in an earlier onset and prolongation of annual stratification phase (Winder and Schindler, 2004; Yankova et al., 2016). Lake warming has also striking impacts on the seasonal mixing regime by weakening deep winter mixing (Woolway and Merchant, 2019), a key process in large deep temperate lakes which ensures oxygenation of the hypolimnion (Schwefel et al., 2016) and upwelling of nutrient-rich deep waters (Salmaso et al., 2014; Schwefel et al., 2019). The insufficient transport of nutrients, such as phosphorus and nitrogen, from deep to surface zones can strikingly limit primary production during the stratified season (Yankova et al., 2017).

While these ongoing changes in physicochemical properties of lakes may hamper the growth of eukaryotic algae, e.g., cryptophytes and diatoms (Yankova et al., 2017), prokaryotic cyanobacteria seem to profit from the fast structural changes within freshwater ecosystems on a global scale (Huisman et al., 2018). On a species level, cyanobacteria profit from physiological traits making them successful competitors among planktonic phototrophs in rapidly changing systems. Some species (e.g., of the genus *Microcystis*) are directly favored by warmer surface water temperatures, especially in combination with eutrophying nutrient loads (Paerl and Huisman, 2008; Huisman et al., 2018). Other species (e.g., *Planktothrix rubescens*) benefit from stronger thermal stability of the water column and thereby may be affected by changes in nutrient fluxes and stoichiometry (Posch et al., 2012). Notably, their frequent appearance was also documented for nutrient-poor lakes and even systems undergoing multidecadal restoration processes (Jacquet et al., 2005). *P. rubescens* is one of the best-studied cyanobacterial species within the genus *Planktothrix*, which also colonizes large deep lakes (Nürnberg et al., 2003) and forms mass developments (the basionym is *Oscillatoria rubescens*, see AlgaeBase^1^).

This cyanobacterium exhibits a set of physiological traits allowing for successful colonization of lakes, already at times when climate change (Dokulil and Teubner, 2012; Anneville et al., 2015) was not an additional driver for its spreading (Ernst et al., 2009; D’Alelio et al., 2011). *P. rubescens* is hardly grazed by any herbivorous consumer: (i) owing to its filamentous morphology, formed by hundreds of single cells in up to 5 mm long fibers, filter feeders are hindered in the successful uptake of *P. rubescens*. (ii) Due to the intracellular storage of multiple toxic secondary metabolites (Blom et al., 2001, 2003; Kurmayer et al., 2016), the cyanobacterium is harmful for most eukaryotes (including humans). Owing to grazing protection mechanisms, this primary producer is a sink and not a link for nutrients within food webs. *P. rubescens* can regulate its vertical position in the water column by an interplay of gas vesicles to stay buoyant or to rise and an accumulation of carbohydrates to sink (Utkilen et al., 1985). Controlled positioning and migration in the water column is essential, as the cyanobacterium is a low-light-adapted phototroph with photoheterotrophic capabilities (Zotina et al., 2003) and this trait ensures competitive advantage over most eukaryotic algae in light-limited zones (Dokulil and Teubner, 2012). Thus, during the period of thermal stratification, the metalimnetic zone seems to fulfill the requirements for *P. rubescens* to form dense populations (Walsby et al., 2004), as this physically separated zone prevents the entrainment of filaments into the turbulent epilimnion. However, during winter periods of deep mixing, *P. rubescens* may be confronted with unfavorable and especially unstable environmental conditions.

The seasonal development of *P. rubescens* in Lake Zurich (Switzerland) and the underlying physiological traits of the cyanobacterium were intensively studied for more than a decade by Walsby and colleagues (see list of references in Walsby et al., 2006). For this deep temperate lake, it was also documented how dominant *P. rubescens* became within a phototrophic microbial community over several decades (1976–2010; Posch et al., 2012). This long-term succession was, at least partially, attributed to climate-related changes (Yankova et al., 2016). During the last decade, we observed, in addition to sustained lake warming, striking changes of the mixing regime reflected by consecutive years of incomplete winter mixing (Yankova et al., 2017).

According to the standing knowledge, a continued increase of *P. rubescens* would be expected as a result of reduced winter mixing and increased water column stability. Here, we broaden this view by showing that the regrowth of *P. rubescens* can be comparably modest after incomplete winter mixing and that population dynamics also responded to interannual variations in weather conditions with large oscillations of the standing stock. Our analysis is based on a 12-year lasting biweekly (weekly during spring) monitoring campaign (2009–2020), directed to major physical and *P. rubescens-*related biological parameters for the water column. Notably, during this period, we observed two distinctive breakdowns of the cyanobacterium, also in years of weak winter mixing but during which the vernal onset of the metalimnion was disrupted by adverse conditions. Additionally, we recorded strong oscillations in population dynamics, in terms of temporal patterns and spatial location within the water body. This variability occasionally manifested by the formation of anomalous surface blooms during the stratification period. Thus, to elucidate favoring aspects but also downside effects of lake warming and interannual weather conditions on *P. rubescens*, we focused on the nexus of three parameters as decisive environmental factors, namely, deep winter mixing, thermal stratification patterns, and light regime. More specifically, we aimed to: (i) assess the reliability of winter mixing as a predictive descriptor for the size of the vernal starting populations as well as for the following overwintering populations, (ii) investigate the influence of a timely formation of a stable metalimnion for the successful reestablishment of *P. rubescens* after deep winter mixing, and (iii) identify the conditions triggering the atypical entrainment of *P. rubescens* populations into the epilimnion during the stratification period.

By presenting a new set of complex interplays between *P. rubescens* and the physical environment, our results can help to improve predictions on how cyanobacterial dynamics could develop in times of climate change and will respond to interannual weather conditions. Our findings may not only concern Lake Zurich (notably, a drinking water reservoir for >1.2 million inhabitants) but will be of relevance for most other restored lakes with reported *P. rubescens* occurrences.

## Materials and Methods

### Sampling Site and Measurement of Parameters

Lake Zurich, Switzerland, is a prealpine, mesotrophic lake in the temperate zone, with two major basins separated by a natural dam, which is a moraine of the retreating glacier during the last ice age. The upper part (21.7 km^2^, 0.4 km^3^), fed by the Linth River inflow, is shallow (*z*_max_ = 48 m) and has a short water retention time of ∼70 days. The lower part (66.6 km^2^, 3.3 km^3^), herein termed as Lake Zurich, is much deeper (*z*_max_ = 136 m) with a water retention time of ∼1.2 years. It is monomictic and potentially holomictic at the end of cold winters.

All presented parameters were measured at the long-term monitoring site of the Limnological Station Zurich (local *z*_max_ = 125 m), which is close to the position of maximal depth (N 47°17.147′, E 8°35.460′, 406 m above sea level). Profiles for physicochemical and biological parameters were determined on a biweekly basis (weekly during spring periods) since the year 2009 until end of the year 2020. In total, we present the evaluation of 320 profiles over a 12-year period. We used an YSI 6600 (YSI 6600 V2 since 2014; YSI Incorporated, Yellow Springs, OH, United States) multiparameter probe for profiling oxygen concentrations (mg O_2_ L^–1^), water temperature (°C), and conductivity (μS cm^–1^). Since 2014, we installed an additional sensor for phycoerythrin measurements (in RFU, relative fluorescence units). A bbe FluoroProbe (bbe Moldaenke GmbH, Schwentinental, Germany) was used for measurements of *in vivo* total chlorophyll *a* concentration (μg Chl *a* L^–1^; Beutler et al., 2002; Hofmann and Peeters, 2013). In addition, the probe assigns, based on fluorescence fingerprints, the *in vivo* Chl *a* concentration to five algal groups, namely, green algae, diatoms, cryptophytes, blue-green algae (i.e., mainly phycocyanin containing cyanobacteria), and the phycoerythrin-containing cyanobacterium *P. rubescens*. The specific calibration of the bbe FluoroProbe *via* optical fingerprints for the detection of *P. rubescens* was first described by Leboulanger et al. (2002). For the *P. rubescens*-specific calibration of our bbe FluoroProbe, we followed the detailed guidelines in the user manual of the company and the published protocol (Leboulanger et al., 2002). Additionally, bbe Moldaenke GmbH offers a specific calibration for the detection of *P. rubescens* as a standard feature of the bbe FluoroProbe. In this study, we focus only on *in vivo* Chl *a* values of *P. rubescens* and on the cumulative Chl *a* values of the remaining groups.

Both probes were lowered from the research vessel on an automatic winch with a speed of ∼0.1 m s^–1^, from the surface to 123 m depth. Probes recorded values at a frequency of 30 measurements per minute, resulting in ∼500–700 single measurements for each profile. Raw data profiles were interpolated on a basis of 1 m depth interval from 0 to 120 m.

Based on the bathymetric data published in Walsby et al. (1998), we calculated the depth-integrated total amount of *P. rubescens*, in metric tons *in vivo* Chl *a*, over the entire water column for each profile. Specific Chl *a* values in 1 m steps between 0 and 120 m were multiplied with the corresponding lake volume for each depth layer and finally integrated for the entire water column. Notably, the water body down to 120 m depth represents 98% of the water volume of Lake Zurich. Net growth rates *μ* (day^–1^) were calculated using the following eq. 1:

(1) $$\mu =\frac{\ln\left(B_{t\,1}\right)-\ln\left(B_{t\,0}\right)}{\left(t\,1-t\,0\right)}$$

where *B*_t0_ *and B*_t1_ are the total amounts of *P. rubescens*, in metric tons *in vivo* Chl *a*, on two consecutive sampling dates (*t*0 and *t*1), respectively.

We evaluated the reliability of *in vivo* Chl *a* measurements for quantifying total amounts of *P. rubescens* by direct comparison with a dataset based on biovolumes. During the investigation period (2009–2020), the Water Supply Zurich (WVZ) determined biovolumes (mm^3^ L^–1^) of *P. rubescens* populations based on microscopic counts and filament length measurements in Lugol’s iodine fixed samples (Utermöhl, 1958). Samples were taken monthly at the long-term monitoring site (*z*_max_ = 136 m) of the WVZ (at a distance of 3 km to our sampling site). Cumulative biovolumes (cm^3^ m^–2^) of *P. rubescens* were computed for the entire water column and for the depth layers 0–20, 20–40, and 40–135 m. During the years 2009–2011, calculations were based on monthly samples from 14 sampling depths: 0, 1, 2.5, 5, 7.5, 10, 12.5, 15, 20, 30, 40, 80, 120, and 135 m. For the years from 2012 to 2020, samples for the depth layer 0–20 m have been taken monthly with an integrating water sampler (Schröder, 1969), and depth-proportional mixed samples from depths of 20, 30, 40, 60, 80, 100, 120, 130, and 135 m were used for the computation of biovolumes in the deep layers (20–40 and 40–135 m).

Concentrations of particulate phosphorus and orthophosphate were determined on a monthly basis for the entire investigation period (2009–2020) by the WVZ for 15 sampling depths (0, 1, 2.5, 5, 7.5, 10, 12.5, 15, 20, 30, 40, 60, 80, 100, and 120 m). Concentrations of nitrate (NO_3_-N) were measured on a monthly basis for 10 sampling depths (0, 1, 2.5, 5, 7.5, 10, 12.5, 15, 20, and 30 m).

### Measurement of Irradiance

For each sampling occasion, underwater irradiance (*E*_d_) was determined with a spherical underwater quantum sensor (LI-COR Biosciences, Bad Homburg, Germany) at 1 m intervals from the surface to a depth of the threshold value of ∼0.05 μmol photons m^–2^ s^–1^. The underwater sensor measured PAR (photosynthetically active radiation) in the range of 400–700 nm. Based on logarithmically (ln) transformed *in situ* irradiance profiles, we determined the three depths reflecting intrinsic light conditions for the cyanobacterium *P. rubescens*. According to Walsby and Jüttner (2006), we determined the exact depth with *E*_d_ = 0.8 μmol m^–2^ s^–1^ (*z*_comp_), an irradiance at which the uptake of various amino acids was stimulated and favored minimal growth. However, this value is still less than the photosynthetic compensation point for autotrophic growth in light (*E*_d_ = 1.6 μmol m^–2^ s^–1^), i.e., when production and respiration processes of the cyanobacterium should result in a net zero growth. Thus, cyanobacteria at *z*_comp_ will definitely be forced to float up in the water column with the help of intracellular gas vesicles, in order to experience photoautotrophic growth. The depth with an *E*_d_ = 6.5 μmol m^–2^ s^–1^ represents the neutral buoyancy depth (*z*_buoy_), where more than 50% of the *P. rubescens* population will stay buoyant (Walsby et al., 2004). Finally, the depth with an *E*_d_ = 25 μmol m^–2^ s^–1^ (*z*_sat_) delimits saturating light conditions, forcing filaments to rather sink than to stay buoyant (Bright and Walsby, 2000).

For each sampling date, we determined the light attenuation coefficient *K*_d_ (m^–1^) as the slope of the linear regression line between depth and logarithmically transformed ln(*E*_d_) values. For autumn and winter months, linear regressions were computed for the entire ln(*E*_d_) profile. However, dense metalimnetic *P. rubescens* blooms during stratification caused strikingly higher *K*_d_ values in these water layers (Schanz, 1985). Thus, *K*_d_ was based on ln(*E*_d_) values from surface to the depth where a strict linear regression was still found (i.e., usually down to 10–12 m). The thickness (m) of the euphotic zone (*z*_eu_) was computed according to Kalff (2002) by the following eq. 2:

(2) $$z_{\text{eu}}=\frac{\ln\left(100\right)}{K_{\text{d}}}$$

### Determination of the Mixed Layer Depth and Stratification Parameters

Water density (*ρ*) or, strictly speaking, potential density referenced to the lake surface was calculated based on the measured temperature and conductivity profiles. First, salinity (*S*) was calculated from conductivity normalized to 25°C by using a lake-specific multiplicative factor (Wüest et al., 1996) of 0.7999 × 10^–3^ [‰ (μS cm^–1^)^–1^]. Potential temperature (*θ*) was calculated from *in situ* temperature and salinity, and the temperature-dependent potential density (*ρ*_θ_) was calculated according to Chen and Millero (1986). Finally, a haline contraction coefficient of *β* = 0.807 × 10^–3^ appropriate for lakes where calcium carbonate is the main contributor to salinity was used to account for the effect of dissolved solids in density (Wüest et al., 1996):

(3) $$\rho =\rho_{\theta }\,\left(1+\beta \,S\right)$$

The depth of the surface mixed layer (*z*_mix_) was calculated as the depth below which water density exceeded a reference near-surface density by 0.01 kg m^–3^. The reference surface density was calculated as the average density in the top 2 m. This was done to avoid the effects of transient near-surface stratification during hours of solar heating, which could result in underestimation of the maximum daily mixing depth, particularly during winter months. The intensity of density stratification was quantified with the water column stability.

(4) $$N^{2}\,\left(z\right)=-\frac{g}{\rho }\,\left(\frac{\partial \rho }{\partial z}\right)$$

where *g* is the gravitational acceleration and *N* is referred to as the buoyancy frequency. The vertical thickness of the metalimnion was characterized with its upper and lower boundaries, defined as the depths where the local stability equates half the maximum *N*^2^ value of the corresponding profile, which defines the center of the metalimnion. We considered that a metalimnion was properly established if the *N*^2^ maximum was larger than 2 × 10^–4^ s^–2^ and its thickness was smaller than 20 m. Those values were chosen according to the specific stratification dynamics of Lake Zurich.

### Meteorological Data

Daily values of meteorological variables (air temperature, relative humidity, atmospheric pressure, wind speed, and solar radiation) throughout the monitoring sampling period (2009–2020) were obtained from the Wädenswil Meteorological Station managed by MeteoSwiss.^2^ The station is located in the southwestern shore of Lake Zurich (47.220958°N, 8.677706°E, 485 m above sea level). The above listed parameters and lake surface temperature (measured in 1 m depth) were recorded daily by a weather station in Mythenquai (operated by the Water Police Zurich) at the northwestern shore of the lake. Occasional data gaps in the Wädenswil data records were filled with information derived from the Mythenquai station. Based on the entire dataset, heat fluxes across the lake surface were calculated following the Monin–Obukhov similarity theory, for latent and sensible heat fluxes (Zeng et al., 1998), and the equations described in Fink et al. (2014), for radiative longwave and shortwave fluxes. The multiplicative calibration constant in the longwave absorption equation was fitted to ensure consistency between the computed net heat flux and the temporal evolution of lake heat content in the monitoring temperature time series. The value of the fitted constant, *a* = 1.02, was consistent with the value found for Lake Constance (Fink et al., 2014). The Python codes for the surface flux calculations are available at https://www.datalakes-eawag.ch/datadetail/452.

### Statistics

Statistical analyses were performed using R (R Core Team, 2019). Parameters such as the depths of *P. rubescens* Chl *a* maximum, of maximal stratification (*N*^2^), and of the three relevant irradiances did not follow a normal distribution (Shapiro–Wilk normality test). Thus, non-parametric tests were applied (Spearman’s rank order correlation) using the R package “corrplot” (Wei and Simko, 2017). A non-parametric seasonal Mann–Kendall tau test (SMK) was applied to test for a monotonic trend over time in the depth-integrated amounts of *in vivo* Chl *a* using the R package “EnvStats” (significance level *α* = 0.05; Millard, 2013).

## Results

### Winter Mixing Dynamics Affect Chemical and Biological Parameters

**FIGURE 1:**
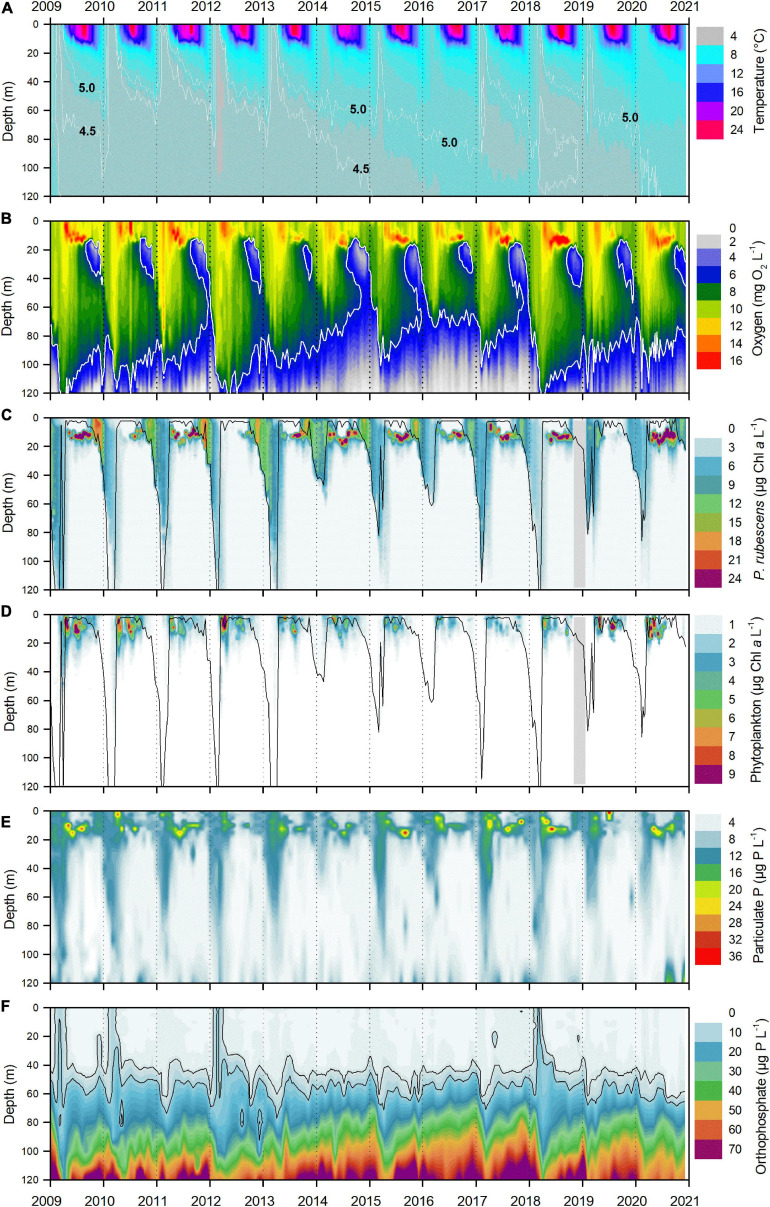
Trends (2009–2020) in thermal stratification, oxygen concentration, phytoplankton chlorophyll values, and phosphorus concentrations in Lake Zurich (Switzerland). **(A)** Water temperature with two white isotherms indicating 4.5 and 5.0°C, respectively. **(B)** Oxygen concentration (mg O_2_ L^– 1^) with white isoline indicating 6 mg O_2_ L^– 1^ (see text for details). **(C)** *In vivo* chlorophyll *a* concentration (μg Chl *a* L^– 1^) of the cyanobacterium *Planktothrix rubescens*. Gray area: no measurements from November 2018 to January 2019. **(D)** *In vivo* chlorophyll *a* concentration (μg Chl *a* L^– 1^) of total eukaryotic phytoplankton. Gray area: no measurements from November 2018 to January 2019. Black lines in panels **(C,D)** indicate the surface mixed layer (*z*_mix_). **(E)** Concentrations of particulate phosphorus (μg P L^– 1^). **(F)** Concentrations of orthophosphate (μg P L^– 1^) with two black isoline indicating 5 and 10 μg P L^– 1^, respectively. Panels **(A–D)** are based on weekly/biweekly profiles (*n* = 320) of parameters, measured with multiparameter probes and interpolated in 1 m steps between 0 and 120 m depth (*n* = 38,420). Panels **(E,F)** are based on monthly analyses (*n* = 144) of 15 sampling depths between 0 and 120 m (*n* = 2,160).

**FIGURE 2:**
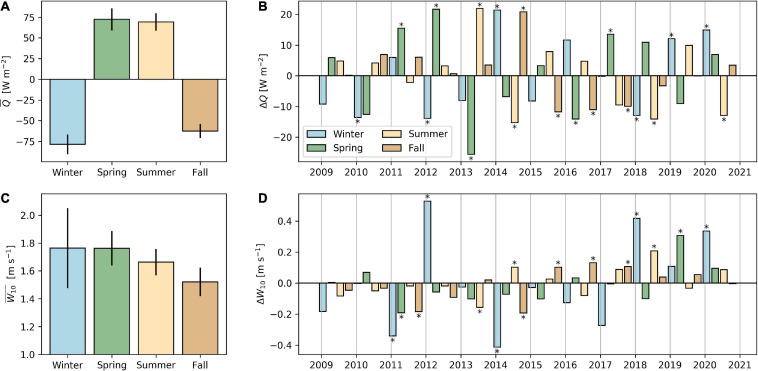
Average (+/– one standard deviation) seasonal net surface heat fluxes **(A)** and wind speed **(C)** during the sampling period 2009–2020, and seasonal anomalies with respect to the long-term seasonal average **(B,D)**. In panels **(B,D)**, seasonal anomalies greater/smaller than +/– one standard deviation of the long-term average are marked with an asterisk (*). Seasons are defined as winter (DJF), spring (MAM), summer (JJA), and fall (SON). Positive heat flux means a net heat gain by the lake.

During winter periods from 2009 to 2013, water temperature at the surface cooled to 4.0–4.5°C, resulting in isothermal conditions over the entire water column (Figure 1A). Thus, a 5-year series of holomixis events (complete water turnover) was recorded (*z*_mix_, black line in Figures 1C,D). In this period, water temperature in the deep hypolimnion (>60 m depth) remained below 5°C even during summer stratification. In the following 3 years (2014–2016), a series of incomplete deep winter mixing went in parallel with a gradual warming of the hypolimnetic zone (see deepening of the 4.5 and 5°C isotherms in Figure 1A) and *z*_mix_ reached only 47, 82, and 61 m, respectively. Thereafter, two winters with complete turnover were observed, followed by incomplete deep mixing in 2019 (*z*_mix_ = 81 m) and 2020 (*z*_mix_ = 86 m). A recent, sudden and striking warming of the deep hypolimnion (Supplementary Figure 1) was observed in February 2020. We attribute this fluctuating temperature increase to flushing of deep water by lateral inflows during intensive storms events (winter 2020 was windier than average; Figure 2D; see also http://meteolakes.ch/#!/hydro/zurich). The combination of both intense cooling from surface heat flux (i.e., a negative heat flux anomaly; Figures 2A,B) and wind activity during winter (Figures 2C,D) appeared as decisive for the strength of deep convective winter mixing (e.g., holomixis in 2012 and 2018). In contrast, years with exceptional weak winter mixing (e.g., 2014 and 2016) were characterized by lower wind activity and less heat loss during winter.

Decreasing hypolimnetic oxygen concentrations clearly reflected the phase of incomplete deep winter mixing between 2014 and 2016. Although *z*_mix_ indicated again deep mixing in 2017 (*z*_mix_ = 115 m, black line Figure 1C), this particular oxygen replenishment was not sufficient to compensate for the apparent deficit which had developed below 80 m depth over several years before (Figure 1B). It was the holomixis in 2018 that led to a full oxygenation of the entire water column again. Historically, several studies described oxygen concentration as the best parameter to characterize the depth and intensity of turnover dynamics in Lake Zurich. Örn et al. (1981) defined a concentration of >6 mg O_2_ L^–1^ as a proxy for *z*_mix_. This empirical value still proved an adequate descriptor for the depth of water turnover (see white isoline in Figure 1B), however, less for the year 2017.

For all, except 3 years (2010, 2012, and 2019), *P. rubescens* formed a dense layer in the metalimnetic zone during summer thermal stratification (Figure 1C). However, in 2012 and 2019, *in vivo* Chl *a* concentrations were extraordinarily low during summer months indicating two distinctive breakdowns in the long-term population dynamics. *P. rubescens* concentrations during winter months perfectly reflected the intensity of water turnover, showing the weak entrainment of filaments into the hypolimnion for the winters 2014–2016 (Figure 1C). *In vivo* Chl *a* concentrations of eukaryotic phytoplankton peaked solely in the epilimnetic zone and during the warm periods in each year of investigation (Figure 1D). Notably, maximal *in vivo P. rubescens* Chl *a* concentrations were up to three times higher than peak concentrations of eukaryotic phytoplankton. This highlights the quantitative dominance of cyanobacteria in the phototrophic microbial community of Lake Zurich during the study period. The large biomass contribution of *P. rubescens* was also mirrored by concentrations of particulate phosphorus (Figure 1E). This parameter, used as a quantitative descriptor for the total organismic biomass, reflected primarily population dynamics of *P. rubescens* in Lake Zurich. Each year, we observed increasing orthophosphate concentrations in parallel with oxygen depletion in the hypolimnetic zone below 60 m (Figure 1F). A clear orthophosphate enrichment of surface zones during water turnover was only obvious in four springs (2009, 2010, 2012, and 2018).

### Population Size in Different Water Strata

**FIGURE 3:**
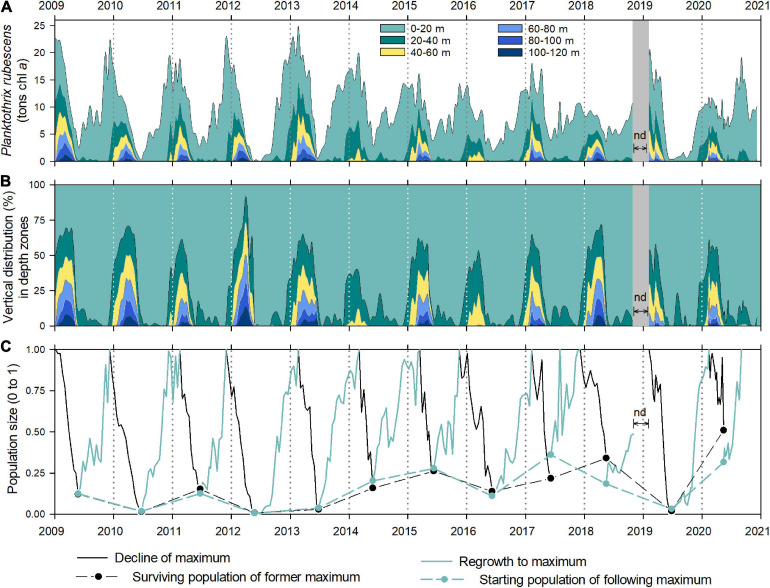
Total population dynamics and vertical distribution of the cyanobacterium *P. rubescens* in Lake Zurich from 2009 to 2020 based on *in vivo* chlorophyll *a* concentrations. **(A)** Cumulative population size (in metric tons chlorophyll *a*) shown in 20 m layers (for the zone of 0–120 m) to highlight the entrainment of filaments into the deep zone during winter mixing. **(B)** Data as in **(A)** but as percentages on the total chlorophyll *a* mass. **(C)** Ratios (0–1) of the surviving population after deep winter mixing to the former maximum population (max = 1; black lines and dots) and ratios of this surviving population to the following maximum population (max = 1; green lines and dots).

The depth-integrated population dynamics of *P. rubescens* gave a complementary picture for cyanobacterial succession patterns compared with the volumetric *in vivo* Chl *a* concentration (compare Figure 1C with Figure 3A). Although dense layers with the highest volumetric concentrations were observed during summer stratification phases (Figure 1C), depth-integrated total population did not peak in these periods (Figure 3A). Instead, total populations increased in late summer/early autumn reaching maximum values in winter months (December–February). From December on, considerable parts of total populations were mixed into deeper water layers (below 20 m, Figure 3B), reflecting the expansion of the mixing layer. In all except 1 year (2012), the majority of “biomass” (∼66–100%) was still found in the upper 60 m and only a minor part was detected in the deepest zone below 100 m (∼0–8%). Notably, the entrainment of filaments below 100 m led to the collapse of gas vesicles of even the strongest genotype GV3 (Bright and Walsby, 1999); thus, cyanobacteria lose their ability for upward migrations. Only in 2012, deep water mixing caused a striking entrainment of filaments into deeper zones and 50% of the population was found below 60 m depth (Figure 3B). In contrast, during incomplete deep winter mixing, especially in 2014 and 2016, the entire population (100%) was concentrated between 0 and 60 m depth. During the years of investigation, minimal cumulative populations were observed after particularly deep winter mixing periods in May to June, with the lowest value recorded in 2012 (Figure 3A), when Chl *a* concentrations were at the limit of detection of the bbe FluoroProbe. Although deep water turnover seemed to restrict the survival of cyanobacteria in some springs (e.g., 2010 and 2012), there was no statistically significant relation between vernal *z*_mix_ and the population minima observed in the following early summer (Spearman’s *ρ* = -0.30, *p* = 0.34, compare Figure 1C with Figure 3A). In the years 2017 and 2018, after deep winter mixing was observed, the “early summer populations” were in the same range as in the 3 years before, when periods of weak winter mixing were recorded. An opposite pattern appeared in 2019, as a complete breakdown of the cyanobacterial population followed an incomplete winter mixing. In addition, the intensity of deep mixing (*z*_mix_) did not allow for predictions on the cyanobacterial growth success during the rest of the year. For the entire dataset, the cumulative population size showed a significant negative trend over time (SMK revealed tau = -0.11, *p* = 0.03).

### Annual Decline and Regrowth of the Population

For each year, we computed the fraction of the surviving population after winter deep mixing (black lines and dots in Figure 3C) and the ratio between this initial vernal population and the following winter maximum (green lines and dots in Figure 3C). In general, there was an astonishing synchrony between these two values in several years. To give one example, in spring 2010, the overwintering population represented 1.6% of the former winter maximum. This surviving population represented 1.8% of the following maximum in winter 2010/2011; thus, the population completely recovered to the same amount as the year before. In contrast, vernal minima were modest from 2014 to 2018, indicating that major parts of winter populations (14–34%) had survived the winter mixing period (Figure 3C). The highest percentage of the overwintering population was recorded in May 2020 with 50%.

**FIGURE 4:**
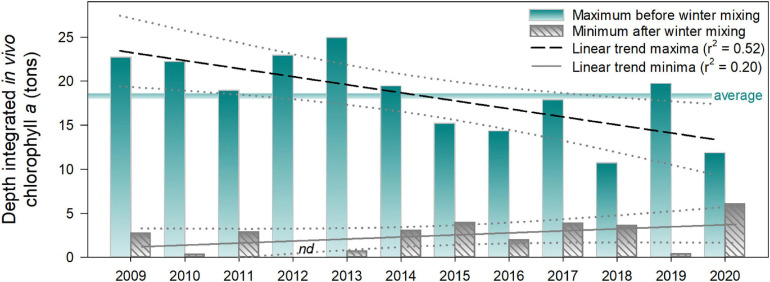
Annual maximal and minimal values of depth-integrated *P. rubescens in vivo* chlorophyll *a* (tons) in Lake Zurich from 2009 to 2020. Trends are shown as linear regressions. Years refer to the time of vernal deep mixing and the subsequent population minimum. Maxima refer to the highest population value between two minima. Maximal values were occasionally reached shortly before deep water mixing of the following year (see high-resolution dynamics in Figure 3A). In 2012, the vernal minimum was at the limit of detection of the bbe FluoroProbe (indicated by *nd*). The horizontal line indicates the average of maxima (18.4 tons) during all the years of investigation.

In summary, we observed in all years a reestablishment of *P. rubescens*, and regrowth was strongest in years with the lowest vernal populations (e.g., in 2010, 2012, 2013, and 2019; Figure 4). On average, the “standing stock maxima” during winter months reached 18.4 metric tons of *in vivo* chlorophyll *a*, with the highest value in winter 2012/2013 (25.0 metric tons) and the lowest in winter 2017/2018 (10.7 metric tons). We observed a decrease of population maxima during the 12 years of investigation (linear regression, *r*^2^ = 0.52), while the vernal minimal values slightly increased (linear regression, *r*^2^ = 0.20; Figure 4).

### Comparison of *in vivo* Chl *a* Measurements With Biovolume Data

For the whole investigation period, we compared depth-integrated *in vivo* Chl *a* values (metric tons) with depth-integrated biovolume (cm^3^ m^–2^) data (Supplementary Figure 2). To our knowledge, there is no systematic study on the average Chl *a* content per biovolume for *P. rubescens*. Kromkamp et al. (2001) reported values between 48 and 85 ng Chl *a* mm^–3^ for *P. rubescens* from Lake Zurich; however, these values were determined during experimental manipulations and seemed to be unrealistically low. Our own singular measurements indicated values of 1.9–5.2 μg Chl *a* mm^–3^ (data not shown). Thus, we see a direct conversion of specific *in vivo* Chl *a* to biovolume with caution, as the Chl *a* content per filament may change over the season but also over depth. However, it was encouraging to find a high synchrony between the two datasets (Supplementary Figure 2). One should keep in mind that an equivalent dataset solely based on microscopic analyses would have only been possible with extensive time- and resource-consuming effort (320 sampling dates times 120 m depth steps would have resulted in 38,400 microscopic samples). Therefore, we are convinced that for the scope of this study, the bbe FluoroProbe was an ideal tool to elaborate a fine-scale dataset for 12 years.

### The Metalimnion as a “Refuge Habitat” for *P. rubescens*

**FIGURE 5:**
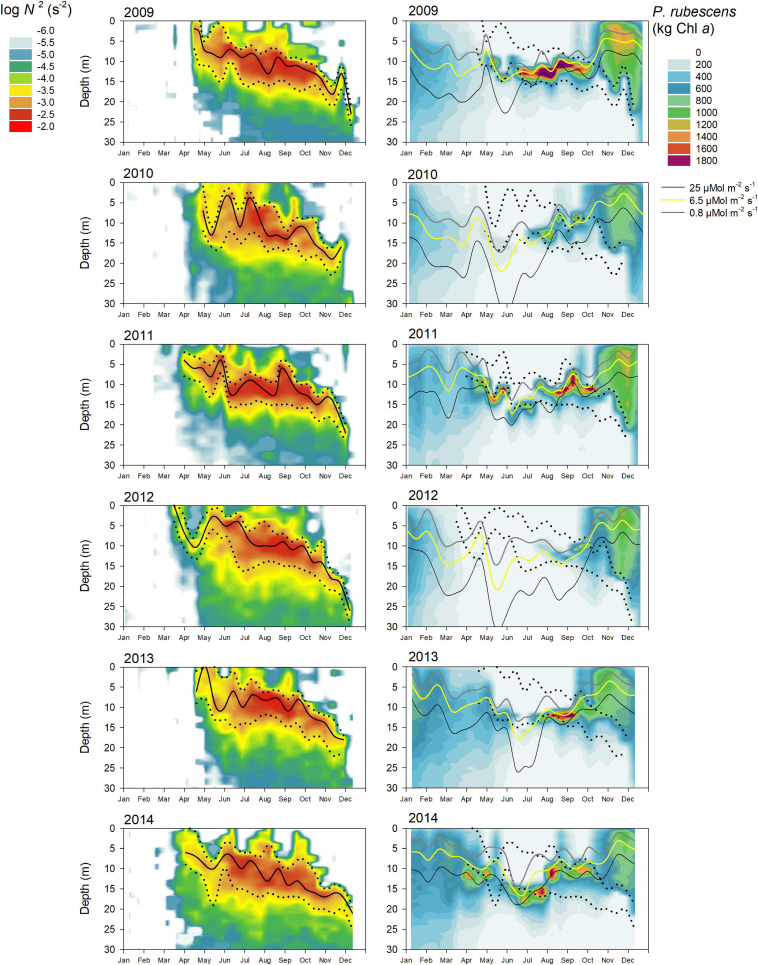
Relationship between water column stability *N*^2^, light intensity, and the cumulative *P. rubescens* biomass for the upper 30 m water column from 2009 to 2014 in Lake Zurich. **Left panels:** seasonal development of the upper and lower boundary of the metalimnion (black dotted line) as well as the depth of the metalimnetic center (black solid line, depth where *N*^2^ reached highest value). **Right panels:** three *P. rubescens* relevant irradiances resulting in neutral buoyancy (*z*_buoy_ where *E*_d_ = 6.5 μmol m^– 2^ s^– 1^, yellow solid line), buoyancy loss (*z*_sat_ where *E*_d_ = 25 μmol m^– 2^ s^– 1^, gray solid line), or buoyancy gain (*z*_comp_ where *E*_d_ = 0.8 μmol m^– 2^ s^– 1^, black solid line). Cumulative *P. rubescens* biomass (kg *in vivo* chlorophyll *a* per 1 m depth step) and the upper and lower boundary of the metalimnion (black dotted lines). All data are based on biweekly measurements in 1 m depth intervals. The exact depths of the three intrinsic irradiance values were calculated from PAR profiles in 1 m depth intervals from surface down to the depth with an irradiance of >0.05 μmol m^– 2^ s^– 1^.

**FIGURE 6:**
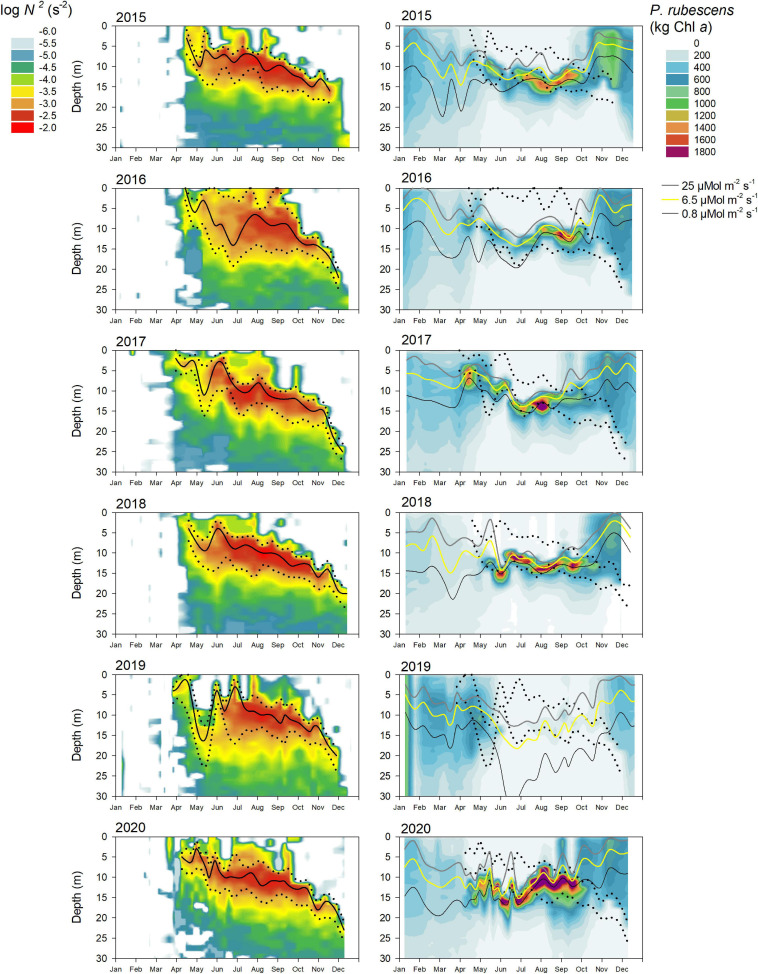
Relationship between water column stability *N*^2^, light intensity, and the cumulative *P. rubescens* biomass for the upper 30 m water column from 2015 to 2020 in Lake Zurich. See legend of Figure 5 for further details.

**FIGURE 7:**
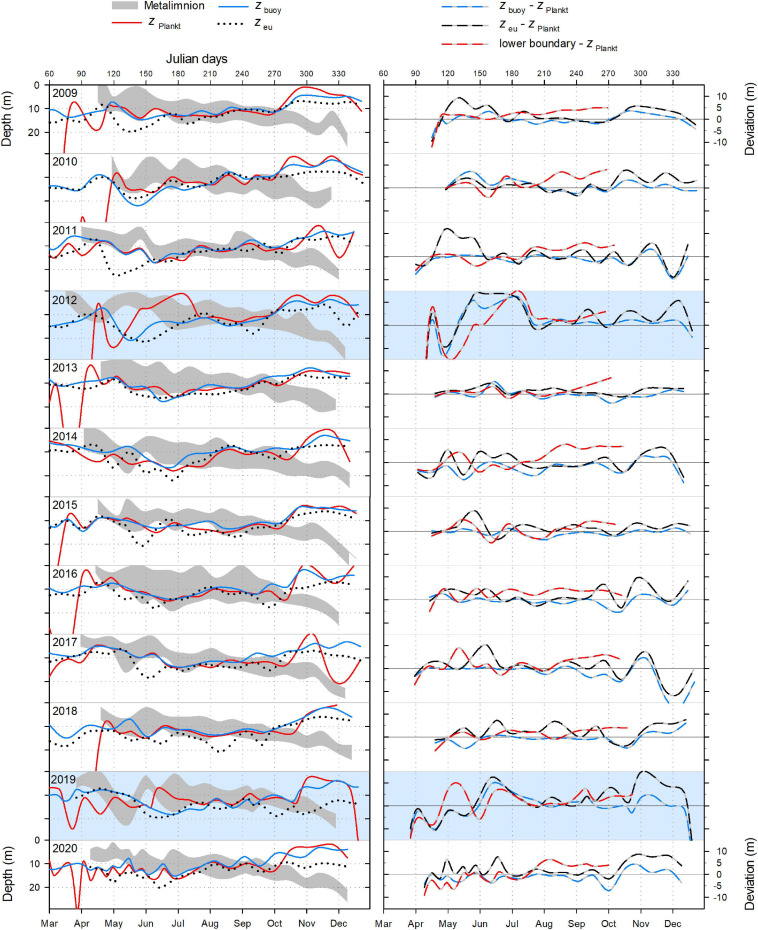
**Left panels:** Metalimnion expansion, the depth of *P. rubescens* chlorophyll *a* concentration maximum (*z*_Plankt_), the thickness of the euphotic zone (*z*_eu_), and the neutral buoyancy depth (*z*_buoy_) in Lake Zurich from 2009 to 2020. **Right panels:** Congruence between *z*_Plankt_ and *z*_buoy_, the lower boundary of the metalimnion, and *z*_eu_. Lines are based on subtraction of the depth of interest minus *z*_Plankt_. Positive deviations (m) indicate that the *P. rubescens* maximum was found less deep than the parameter of comparison, and vice versa for negative deviations (m). Note the striking incongruence between parameters during spring 2012 and 2019 (years are indicated by a blue background).

During most years, *P. rubescens* formed a dense layer in the metalimnetic zone during strong thermal stratification. However, the metalimnion in Lake Zurich appeared from year to year as a highly variable habitat in terms of temporal and spatial dynamics (left panels in Figures 5–7). We observed striking differences concerning the timing of formation, the spatial expansion at the start phase, and the thickness of the metalimnion during the annual succession. In general, stable thermal stratification started at the beginning of April, and there was a constant deepening of the metalimnetic zone in parallel with an increasing warming and expansion of the epilimnion. By comparing the characteristics of the metalimnion (center with maximum *N*^2^, vertical extension) with the annual successions of *P. rubescens* (right panels in Figures 5, 6), the following trends were recorded: (i) maximal cyanobacterial concentrations were not located in the metalimnion center. (ii) From May to at least August, cyanobacteria accumulated rather at the lower boundary of the metalimnion (right panels in Figure 7). (iii) In September/October, we observed upward translocations of the cyanobacterial layer toward the turbulent epilimnetic zone (Supplementary Figure 3). (iv) During the further deepening of the metalimnion in November/December, the cyanobacterial population migrated into the turbulent surface layer in parallel with increasing total population sizes (compare with Figure 3A).

In summary, the metalimnion served as a “refuge” during summer, ensuring that filaments were not entrained into the turbulent epilimnetic zone. Serious anomalies in the annual succession of *P. rubescens* were observed for 2014 and 2020. In both years, parts of the metalimnetic populations were entrained into the turbulent epilimnetic zone already at the beginning of September. As entrainment took place during the still ongoing bathing season, these situations gave a cause for serious concern and asked for special measures at those times (see section “Discussion” for details).

### Exact Positioning of *P. rubescens* Triggered by the Light Regime

For all, except 2 years (2012 and 2019), there were significant correlations (Table 1) between the depth of *P. rubescens* maxima and depths with intrinsic irradiance values (lines in right panels of Figures 5, 6). During thermal stratification, the cyanobacterial layer was largely concentrated between the upper boundary (*z*_sat_ where *E*_d_ = 25 μmol m^–2^ s^–1^, saturating light condition) and the lower boundary (*z*_comp_ where *E*_d_ = 0.8 μmol m^–2^ s^–1^) of the relevant light climate. For most years, the location of population maxima was strongly correlated with the neutral buoyancy depth (*z*_buoy_ where *E*_d_ = 6.5 μmol m^–2^ s^–1^), where in theory more than 50% of the population should stay buoyant (right panels in Figure 7). The computation of *z*_eu_ can be used as a crude approximation to predict the depth of the *P. rubescens* layer. However, correlation coefficients were less significant (Table 1).

**TABLE 1:** Correlation coefficients (Spearman’s correlation) for the depth of *Planktothrix rubescens* Chl *a* maxima versus the depth for three intrinsic light conditions, the upper and lower boundary of the metalimnion, and the depth of maximal stratification.

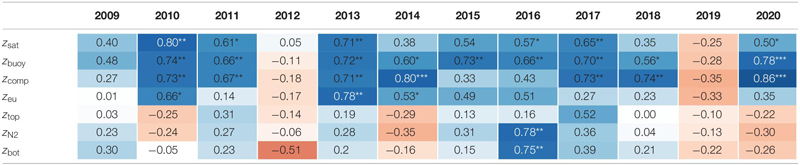

z_sat_, depth of saturating irradiance; z_buoy_, depth of irradiance for neutral buoyancy; z_comp_, depth of irradiance below photosynthetic compensation point but stimulating heterotrophic uptake of amino acids; z_eu_, depth of euphotic zone; z_top_, upper boundary of metalimnion; z_N2_, depth of maximal stratification within the metalimnion; z_bot_, lower boundary of metalimnion. Significance levels: *P < 0.05, **P > 0.01, ***P < 0.001.

*The color gradient indicates the strength and direction of the relationship, red for negative and blue for positive correlations.*

There was a strong impact of dense cyanobacterial layers on the light climate in deeper zones. For the three summers with minimal populations (2010, 2012, and 2019), light penetration increased drastically during the warm period, i.e., *z*_comp_ was found up to 10 m deeper than in years with densely stratified *P. rubescens* populations (Figures 5–7).

### Influence of Metalimnion Formation and Light Conditions

For the year 2019, a paradoxical situation was observed: although a large part of the cyanobacterial standing stock remained until May, a complete breakdown of the population was recorded afterward, and a reestablishment did not happen before November (Figures 5, 6). In the same year, several cooling phases and strong wind events in April and May (spring 2019 was cooler and windier than average; Figures 2B,D) caused a disruption and strong translocation of the stratified water masses, and the establishment of a compact metalimnion was delayed (only from mid-June on). Notably, the breakdown of the cyanobacterial population went in parallel with a striking deepening of the metalimnion center down to 19 m in mid-May. Further cooling phases in June and July caused repetitive variations in the spatial expansion of the metalimnetic zone. For 2019, there was no significant correlation between intrinsic irradiance depths and *P. rubescens* (Table 1).

Based on all years of investigation, the congruence between *z*_buoy_ and the vertical location and extension of the metalimnion seemed to be an intrinsic factor for the successful establishment of *P. rubescens* after winter deep mixing (right panels in Figure 7). The positioning of *z*_buoy_ within or near to the metalimnion during spring appeared as prerequisite for the formation of a cyanobacterial layer in this zone. A striking imbalance between these parameters as observed in 2012 and 2019 was followed by minimal population sizes during the summer months.

### Two Growth Phases of *P. rubescens*

**FIGURE 8:**
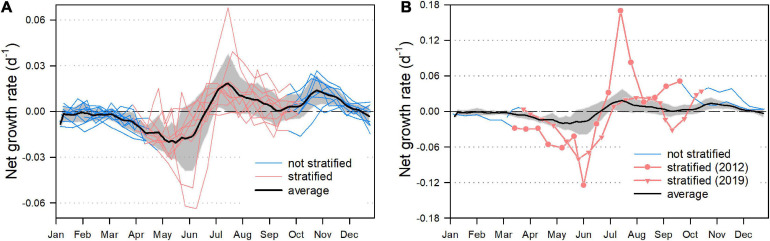
Net growth rate (day^– 1^) of *P. rubescens* (computations are based on cumulative *in vivo* chlorophyll *a* masses presented in Figure 3A). **(A)** Net growth rates (day^– 1^) for the entire study period except for the years 2012 and 2019. Each line represents 1 year of investigation: blue coloration indicates that *P. rubescens* is located in the mixed zone and red coloration indicates the positioning of the cyanobacterium within the stratified metalimnion. Black line: average ± the standard deviation (gray area). **(B)** Net growth rates for the years 2012 and 2019, with the same color code as in **(A)**. Black line and gray area: as in **(A)** for comparison.

For the 12 years of investigation, we computed daily net growth rates, with a special focus whether *P. rubescens* was stratified within the metalimnion or not (Figure 8). From January until the onset of thermal stratification, i.e., when *P. rubescens* was trapped in the mixed water layer, we observed zero or negative net growth rates (Figure 8A). Surprisingly, negative rates were even found for the following period (usually until mid of June) when the cyanobacteria were already stratified within the metalimnion. The first peak in population growth was recorded between mid of June until September. During this period, *P. rubescens* formed a dense layer within the metalimnion. However, a second growth phase was observed from October on, when cyanobacteria already escaped the metalimnion and colonized the turbulent epilimnetic zone. For the 2 years (2012 and 2019) with the most striking breakdowns of the populations, we found quite deviating growth patterns (Figure 8B). In view of the entire dataset, the absolute minimum and maximum net growth rates were recorded in 2012. Exceptional were also the high growth rates from October 2019 on, leading to a reestablishment of the *P. rubescens* population during fall/winter. The autumnal colonization of the epilimnion did not coincide with elevated orthophosphate or nitrate concentrations in this zone (Supplementary Figure 3). In contrast, it seemed that *P. rubescens* caused a further decrease of especially nitrate concentrations during the first period of its epilimnetic expansion.

## Discussion

### *P. rubescens* in Lake Zurich

The first document on the appearance of *P. rubescens* in Lake Zurich dates back to October 8, 1899 (a newspaper article in the Neue Zürcher Zeitung about the “Burgundy-blood phenomenon”). This first mass development of *P. rubescens* led also to a characteristic trace substance in the sediment. Züllig (1981) found the highest concentrations of the carotenoid oscillaxanthin in the sediment layer of 1899. Since then, several authors described annually occurring blooms until the early 1960s (Thomas and Märki, 1949; Thomas, 1969). Interestingly, during the strongest phase of eutrophication (1964–1974), *P. rubescens* biomass was extremely low, even falling below the limit of microscopic detection (Thomas, 1969). In this period, *P. rubescens* likely experienced strong light limitation due to shading effects from mass developments of diatoms and green algae. However, causal reasons for the putative disappearance have not been elucidated during that time. Starting from 1975 on, the cyanobacterium was detected in each year of investigation (Ostermaier et al., 2012), with a continuously increasing contribution to total phytoplankton (Posch et al., 2012) and to the prokaryotic community (Van den Wyngaert et al., 2011). The multidecadal reestablishment of the cyanobacterium went in parallel with rising N:P ratios in the pelagic zone, owing to increasing nitrate (NO_3_) and decreasing orthophosphate concentrations (Posch et al., 2012). *P. rubescens* was most likely favored by this changing stoichiometry, as it requires inorganic N sources (as, e.g., NO_3_) and cannot fix N_2_ directly like other cyanobacterial species.

### Climate Change as a Factor Favoring *P. rubescens*

Since the 1970s, climate change seemed to be an additional driving factor for the persistence of the cyanobacterium in Lake Zurich. Particularly noteworthy is winter warming, which has shown to strengthen the density stratification in various deep temperate lakes around Europe and is expected to increase in the future (Straile et al., 2003; Schwefel et al., 2016; Rogora et al., 2018). Observed consequences are earlier and prolonged thermal stratification phases (Yankova et al., 2016) and incomplete deep winter mixing, conditions reported to be favorable for *P. rubescens* (Walsby et al., 1998; Anneville et al., 2004; Jacquet et al., 2005; Dokulil and Teubner, 2012; Posch et al., 2012). Deep mixing during winter was identified as the intrinsic “natural” mortality factor for these phototrophs, since the entrainment in depths >100 m (critical pressure) leads to the collapse of even the strongest gas vesicles of genotype GV3 (Bright and Walsby, 1999). As *P. rubescens* can rebuild gas vesicles only in light, cyanobacteria forced to deep water >100 m lose their ability for upward migrations. In contrast, filaments above the critical depth maintain their intact gas vesicles (Walsby et al., 1998) and remain viable for weeks even in the cold and dark (Holland and Walsby, 2008). These filaments may migrate back to upper water strata, potentially forming a recruitment for the new metalimnetic population (Walsby, 2005). The recently observed frequent appearance of incomplete deep winter mixing, entraining filaments only to depths of 47–82 m, guaranteed a high starting population for *P. rubescens* in the seasonal successions of several years (e.g., period of 2014–2016, Figures 3A, 4).

### Possible Negative Effects of Climate Change for *P. rubescens*

Surprisingly, the climate-induced reduction in deep winter mixing seemed to be not only of benefit for the annual development of *P. rubescens*. In years following incomplete deep mixing, differences between annual biomass minima and the following maxima in autumn were modest, speaking for only a moderate reestablishment of the total population size (Figures 3C, 4). On the one hand, this may be linked to reduced import of nutrients from the deep into surface zones during incomplete winter mixing (Yankova et al., 2017), thus limiting the growth success of *P. rubescens*. At least for the eukaryotic phytoplankton in Lake Zurich, the vernal lack of essential nutrients (mainly phosphorus) was identified as the main reason for the reduction of mass developments during springtime (Yankova et al., 2017). Nutrient limitation would also be in agreement with studies from Lac du Bourget (France), where the extensive reduction of phosphorus concentration was proposed as a key factor responsible for the breakdown of the former dominant *P. rubescens* populations (Jacquet et al., 2014). On the other hand, deep winter mixing entrains cyanobacterial filaments into deeper water strata with gradually increasing nutrient concentrations. Notably, orthophosphate concentrations steadily increase from a depth of 40 m toward maximal depth in Lake Zurich (Figure 1F). Thus, an enforced deep entrainment above the critical pressure depth may even be beneficial for *P. rubescens*, given that cyanobacteria have the capacity of luxury uptake of nutrients (as net growth was usually negative during that period). The storage of surplus phosphorus during the mixing phase would be advantageous for growth during the following season, i.e., when orthophosphate concentrations are usually below the limit of detection, but NO_3_ is still not limiting (Yankova et al., 2017). There are indications from lab studies on isolated strains that *P. rubescens* may indeed adapt its cellular stoichiometry depending on the available nutrient regime (Frenken et al., 2017). For Lake Zurich, however, a detailed seasonal study on the *in situ* elemental composition of filaments is lacking.

The depth of winter mixing is thus a crucial parameter for the success of *P. rubescens* in Lake Zurich, as it guarantees the accessibility of nutrients on the one hand, but it is deadly for parts of the population when exceeding the critical depth on the other hand. A long-lasting series of strikingly weak winter mixing may thus lead to stronger nutrient limitation not only for eukaryotic phototrophs but also for *P. rubescens*. Cyanobacterial biomass showed indeed a decreasing trend in Lake Zurich for 2009–2020 during which a series of four consecutive years (2014–2017) with incomplete winter mixing was recorded. Additionally, growth patterns in 2020 pointed to nutrient limitation in the further annual succession after the overwintering population was exceptionally large (50%). In that year, the cyanobacterial maximum was already reached in September without further increase during winter 2020/2021 (data not shown).

Our dataset, covering the last 12 years, illustrates that total cyanobacterial biomass reached a certain threshold in Lake Zurich, and we found no statistically significant additional long-term increase. This contrasts the documented long-term rise in population size from the period of 1975 to 2010 (Posch et al., 2012). To date, we can only speculate which factors or resources define the threshold for the current absolute standing stock of *P. rubescens* in Lake Zurich. Annual average concentrations of total phosphorus in Lake Zurich fluctuated only little since the 2000s (Yankova et al., 2017) and could limit additional increase in cyanobacterial biomass. Observations from other lakes indicate that decreasing phosphorus concentrations can ultimately reduce *P. rubescens* biomass (Dokulil and Teubner, 2012; Jacquet et al., 2014). However, a targeted assessment of the minimal concentrations of major nutrients necessary to support *P. rubescens* growth in Lake Zurich is not available at present. For the future, nutrient enrichment experiments could help to identify if phosphorus is the main factor limiting the maximum cyanobacterial biomass in Lake Zurich.

### Influence of Metalimnion Formation and Light Conditions in Spring

Spring is characterized by the onset of thermal stratification and especially susceptible to climate-related changes. For *P. rubescens*, this phase represents the recruitment period of filaments from greater depths to the newly forming metalimnion. Changes of the thermal stratification patterns during this sensitive period can therefore substantially influence population dynamics. In the alpine lake Mondsee (Austria), *P. rubescens* was recorded to profit from climate warming effects during late spring (Dokulil and Teubner, 2012). In Lake Geneva (Switzerland), a large perialpine lake, warmer springs, presumably resulting in earlier onset of stratification, were also observed to benefit cyanobacterial growth (Gallina et al., 2017). The photosynthetic activity of *P. rubescens* is strikingly influenced by even small changes in irradiance (Walsby et al., 2001). Thus, stratification in the zone of the ideal light climate is a prerequisite for a successful vernal survival. In case of cyanobacterial entrainment into the surface zone during spring or early summer due to wind or cold-air outbreaks, they may experience higher than saturating irradiances for potential growth. This does not necessarily mean photoinhibition. For example, Bright and Walsby (2000) described for a *P. rubescens* isolate from Lake Zurich a maximal growth rate of 0.12 day^–1^ at relatively low irradiance levels of *E*_d_ = 25 μmol m^–2^ s^–1^. The cyanobacterium tolerated higher irradiances up to *E*_d_ = 200 μmol m^–2^ s^–1^, however, without any further rise in growth rate. The crucial point for survival of *P. rubescens* during an early epilimnetic entrainment is most probably competition with eukaryotic algae (mainly cryptophytes and small centric diatoms). Although *P. rubescens* could profit from a luxury uptake of nutrients, eukaryotic algae appear to have an advantage due to their higher maximal growth rates during spring. Notably, the maximal growth rates for *P. rubescens* are even the lowest documented within cyanobacteria. In general, the vernal phase stands for harsh environmental conditions, when the interplay of ideal insolation and thermal stratification is decisive for the survival of *P. rubescens* in Lake Zurich and possibly many other deep stratifying lakes. Our long-term data confirm earlier observations (Micheletti et al., 1998; Walsby et al., 2004) showing that, during the first stratification phase, population-based net growth rates are in any case still minimal or below zero. Whether this decrease is also linked to an increased vulnerability toward parasites (viruses or chytrids) has yet to be investigated. It could further indicate a response to environmental stress endured during winter deep mixing. There is increasing knowledge that various cyanobacterial species can undergo programmed cell death triggered by a variety of abiotic stressors (see summary in Aguilera et al., 2021).

### The Metalimnion in Summer: A Refuge and a Zone of Growth for *P. rubescens*

Commonly observed in the metalimnion of stratified lakes during summer, *P. rubescens* might be defined as a metalimnetic species. However, in view of computed growth dynamics for 12 years (Figure 8), this definition seems to hold true only for distinct phases during the seasonal succession. Thomas and Märki (1949) already described the distinct seasonal pattern of *P. rubescens* in Lake Zurich, i.e., that the population is densely stratified in the metalimnion during summer and gets increasingly entrained in the epilimnion toward autumn. The basic patterns of these dynamics are still observable nowadays, despite the changes Lake Zurich underwent by effects of climate warming during the past century.

During summer, the metalimnion serves as a refuge and *P. rubescens* would probably not be able to survive without this safe zone, due to damaging irradiances at the water surface (up to 2,000 μmol m^–2^ s^–1^) and nutrient limitation in the epilimnion resulting from competition with faster growing eukaryotic algae (Walsby et al., 2004). Secondly, the metalimnion also represents the zone for the first peak in population growth. Interestingly, the exact positioning of growing filaments in this temporary habitat was triggered primarily by light climate and not by water column stability (*N*^2^). Here, the neutral buoyancy depth (*z*_buoy_) proved as the best descriptor for the vertical layering of cyanobacteria (Figures 5–7). From an evolutionary point of view, it is of interest (i) whether *P. rubescens* adapted in Lake Zurich to distinct irradiances restricted to the metalimnetic zone and (ii) whether it could adapt to climate-induced changes by spatial expansion of the metalimnion.

There are indications that *P. rubescens* strains show physiological adaptation to intrinsic irradiances in lakes with different metalimnetic light climate. Neutral buoyancy for a strain isolated from Blelham Tarn (English Lake District) was observed at *E*_d_ = 11.7 μmol m^–2^ s^–1^ compared with *E*_d_ = 6.5 μmol m^–2^ s^–1^ for strains from Lake Zurich (Walsby et al., 2004). However, the population was still stratified in the metalimnion, which formed at shallower depths in Blelham Tarn (Davis et al., 2003). On the other hand, a strain from Lake Gjersjøen (Norway) showed neutral buoyancy at a lower value of *E*_d_ = 5 μmol m^–2^ s^–1^ (Walsby et al., 2004).

On a long-term view, lake warming may cause a spatial expansion of the epilimnion and, thus, a deepening and a reduced spatial expansion of the metalimnetic zone in Lake Zurich (Yankova et al., 2016). This change in stratification patterns may bear risks for *P. rubescens*, as filaments will be forced into the turbulent surface zone when the synchrony between ideal irradiance and metalimnion expansion is lost. It is yet an open question, whether cyanobacteria would adapt in time to a lower irradiance for sustaining their neutral buoyancy in a deeper metalimnion.

In terms of ecosystem services, the situation gets critical when *z*_buoy_ is above the metalimnion during the summer recreational season (see year 2020 in Figure 6). During the warmest months, July and August, swimmers using the lake for recreation do not get in direct contact with high concentrations of *P. rubescens*. This statement seemed to be true during the last three decades; thus, many bathers were even not aware of harmful cyanobacteria in Lake Zurich. However, owing to increasing air and surface water temperatures also in autumn, the bathing season extended over the last decades. It happened in the first days of September 2020 that environmental authorities had to warn the public about lake areas with red surface scums. The acceptance of this new measure was moderate, firstly as the public was not yet sensitive to this topic, and secondly, surface scums showed temporal and local dynamics, thus not being obvious on a lake wide view for everyone. As further extensions of the bathing season are expected, such critical epilimnetic entrainments will most probably increase in the future. Frequent determination of *z*_buoy_ combined with temperature profiles appears as a suitable predictive factor to recognize these critical situations in time.

### The Autumnal Growth Phase of *P. rubescens* in the Epilimnion

With decreasing light intensities toward autumn, it seems that the metalimnion is no longer needed as a safe zone for *P. rubescens*. Jacquet et al. (2005), for example, showed for Lac du Bourget that the *P. rubescens* population entered the epilimnion in autumn at times when stratification was still persistent. For Lake Zurich, we documented a similar dynamic of the *P. rubescens* population. In all years of our investigation, most of the population was entering the epilimnion at times when a metalimnion was still identifiable. This spatial relocation occurred in parallel with decreasing depths of intrinsic irradiances (Figures 5–7). Despite being capable of positive net photosynthesis at irradiances as low as *E*_d_ = 1.6 μmol m^–2^ s^–1^, *P. rubescens* is forced upward to maintain positive net production in autumn (Bright and Walsby, 2000). Therefore, epilimnetic entrainment in autumn may not entirely be forced by deepening *z*_mix_ but also driven by light-dependent buoyancy regulation. Recently, the role of convection-induced turbulences was highlighted, which may break up the strong stratification in autumn, allowing *P. rubescens* to escape the metalimnion and to follow specific irradiances (Fernández Castro et al., 2021).

In each year of our investigation, the relocation of *P. rubescens* into the turbulent zone was followed by a second peak in net growth rates (after the first metalimnetic in summer, Figure 8) and an increase in total population size (Figure 3), usually lasting until the end of the year. However, it did not coincide with higher orthophosphate and nitrate concentrations in this zone (Supplementary Figure 3). Epilimnetic water temperatures during this period ranged from 10 to 18°C (Figure 1A) and met conditions observed in the deeper zone of the metalimnion where *P. rubescens* accumulates during summer. Temperatures as low as 10°C proved sufficient to support cyanobacterial growth (Bright and Walsby, 2000). Therefore, we attribute the autumnal increase in biomass mainly to the release of light limitation. Toward the end of summer stratification, *P. rubescens* usually forms very thin and dense metalimnetic layers resulting in heavy self-shading and potential intraspecific competition. Autumnal surface circulation improves the accessibility to sufficient light conditions for the whole population, and in combination with a mobilization of stored nutrients, as already documented for *Planktothrix* spp. (Hampel et al., 2019), it could explain the observed renewed growth. Notably, while epilimnetic light and temperature conditions in autumn are favorable for *P. rubescens*, eukaryotic algae reach their minima during this period (Figure 1D). This further benefits *P. rubescens* by minimizing interspecific competition in the epilimnion. Our long-term observations fully confirm previous studies documenting this autumnal growth phase for single years in Lake Zurich (Micheletti et al., 1998; Bright and Walsby, 2000; Walsby and Schanz, 2002; Van den Wyngaert et al., 2011). The bimodal growth dynamics of *P. rubescens* were also described for Lake Mondsee (Austria) in a long-term study during 25 years (Dokulil and Teubner, 2012). For some lakes experiencing seasonal ice coverage, it was even observed that *P. rubescens* continues to grow in winter while accumulating directly under the ice cover (Halstvedt et al., 2007; Lenard, 2015). The underlying mechanisms for these under-ice blooms appear to be light-driven as well. Ice and additional snow coverage can drastically reduce light in the epilimnion, leading to favorable conditions for low-light-adapted species such as *P. rubescens* (Wright, 1964; Lenard, 2015). Notably, in such lakes, climate change can also be expected to negatively affect *P. rubescens* blooms by shortening winter ice coverage. In summary, all studies documented that positive growth was also achieved in the turbulent epilimnion during autumn or even in calm conditions just below the ice during winter. Thus, the classification of *P. rubescens* as a “strict metalimnetic species” holds true only for distinct periods during the year.

## Conclusion

Our study highlights that the effect of secular lake warming on *P. rubescens* was not as unidirectional as previously assumed for the last decade. Intensive deep winter mixing and repetitive disruptions of the vernal metalimnion structure owing to strong winds both resulted in a massive decrease of the population but pronounced regrowth during thermal stratification. Weak convective deep mixing was linked to lower wind activity and less heat loss during winter and caused high survival ratios of overwintering cyanobacterial populations (up to 50%). However, compared with years with distinct population breakdowns, high start populations after incomplete winter mixing showed (i) surprisingly moderate regrowth during the stratified season and (ii) below average population maxima. Overall, a long-lasting series of strikingly weak winter mixing may cause unfavorable conditions for *P. rubescens*, likely as a result of nutrient depletion. A special focus should be directed on extreme weather events, which disturb annual stratification patterns and, in consequence, the growth success of *P. rubescens* in lakes. On a long-term view, it is of interest whether *P. rubescens* may adapt to climate-induced disharmonies between metalimnion expansion and ideal light conditions.

## Data Availability Statement

The datasets presented in this article are not readily available because specific data can be obtained from the authors by request. Requests to access the datasets should be directed to corresponding author TP, posch@limnol.uzh.ch.

## Author Contributions

Conduction of sampling campaigns and data collection were done by DM, EL, and TP during 2009 to 2020 (Also by DK since 2018). TP, EL, and DK contributed to the design of the monitoring program. All authors contributed to the article, data synthesis, completion of the manuscript was a collaborative project, and approved the submitted version.

## Conflict of Interest

The authors declare that the research was conducted in the absence of any commercial or financial relationships that could be construed as a potential conflict of interest.

## Publisher’s Note

All claims expressed in this article are solely those of the authors and do not necessarily represent those of their affiliated organizations, or those of the publisher, the editors and the reviewers. Any product that may be evaluated in this article, or claim that may be made by its manufacturer, is not guaranteed or endorsed by the publisher.

## Footnotes

1
http://www.algaebase.org/search/species/detail/?species_id=30299

2
http://www.meteoswiss.ch
