# Supplementary material for: The Red Harmful Plague in Times of Climate Change: Blooms of the Cyanobacterium Planktothrix rubescens Triggered by Stratification Dynamics and Irradiance
Source: Front Microbiol. 2021 Aug 25;12:705914. doi: 10.3389/fmicb.2021.705914 (PMC8425285; doi:10.3389/fmicb.2021.705914)
Supplement: Supplementary file 1 [file Data_Sheet_1.PDF]

Supplementary Information (Figure S1, S2, S3) to:

The red harmful plague in times of climate change: Blooms of the cyanobacterium *Planktothrix rubescens* triggered by stratification dynamics and irradiance

Deborah Knapp[1], Bieito Fernández Castro[2], Daniel Marty[1], Eugen Loher[1], Oliver Köster[3], Alfred Wüest[4,5], Thomas Posch[1]*

[1] Limnological Station, Department of Plant and Microbial Biology, University of Zurich
Seestrasse 187, CH-8802 Kilchberg, Switzerland

[2] Ocean and Earth Science, National Oceanography Centre, University of Southampton, SO14 3ZH, Southampton, UK

[3] Zurich Water Supply, Hardhof 9, CH-8021 Zurich, Switzerland

[4] Physics of Aquatic Systems Laboratory, Margaretha Kamprad Chair, Institute of Environmental Engineering, École Polytechnique Fédérale de Lausanne, CH-1015 Lausanne, Switzerland

[5] Eawag, Swiss Federal Institute of Aquatic Science and Technology, Surface Waters – Research and Management, Seestrasse 76, CH-6047 Kastanienbaum, Switzerland

* corresponding author:
Thomas Posch
Phone: 0041 44 63 492 24
Email: posch@limnol.uzh.ch

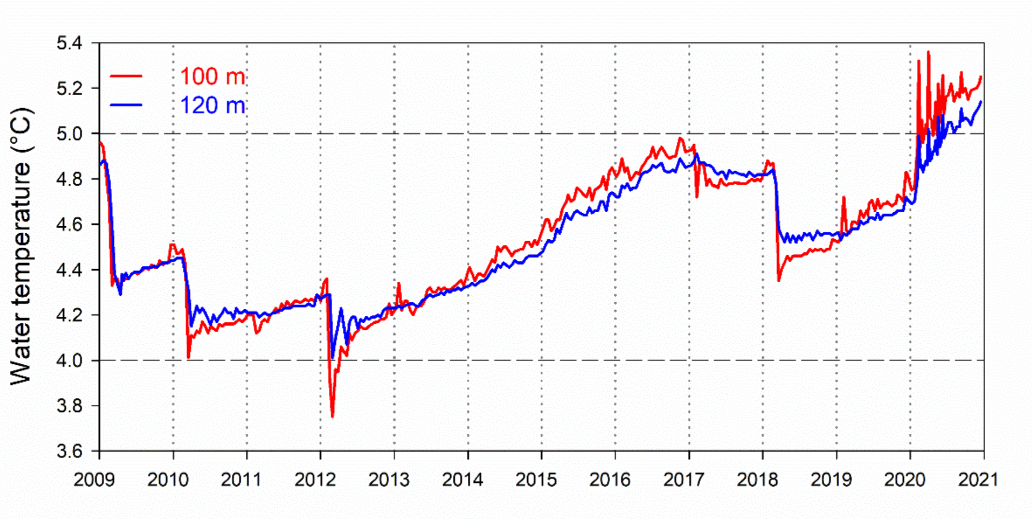

Supplementary Figure S1: Hypolimnetic water temperatures for two selected depths (100 m, 120 m) in Lake Zurich (z_{max} = 135 m), Switzerland, during January 2009 to December 2020. Measurements were done on a biweekly basis (weekly during spring, in total 320 samplings). A YSI 6600 (YSI 6600 V2 since 2014) multiparameter probe was used for the determination of water temperature (°C). Note the cooling effect of the deep water turnover in 2012, and the continuous increase in water temperature during the period of 2014 to 2017. That period was characterized by a consecutive series of incomplete winter mixing. A sudden temperature increase to values above 5 °C was recorded in winter 2019 / 2020 after intensive storms (see Figure 2). Temperature profiles for the entire water column and depths of winter mixing are shown in Figure 1.

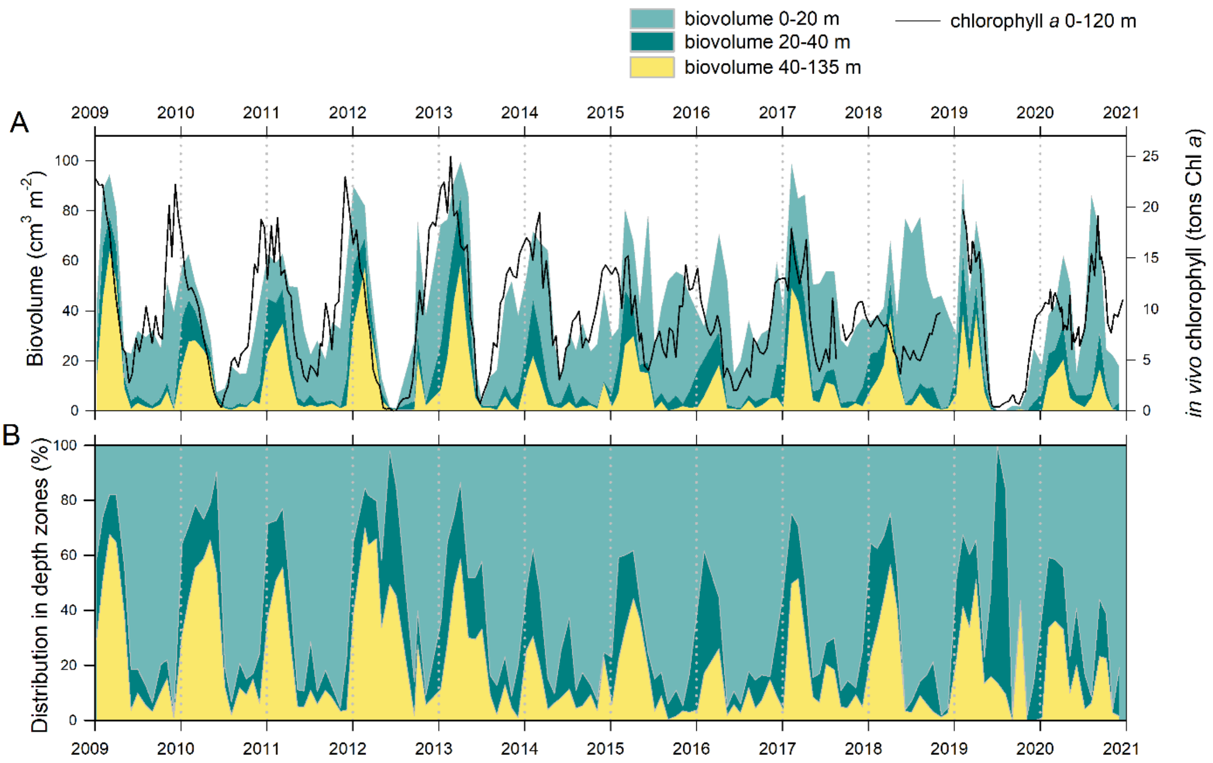

Supplementary Figure S2: (**A**) Comparison of depth-integrated total amounts of *Planktothrix rubescens* specific *in vivo* chlorophyll *a* (tons Chl *a*) with total amounts of *P. rubescens* based on biovolumes (cm^3 m^{-2}) during the investigation period (2009 to 2020). *In vivo* Chl *a* concentrations (µg Chl *a* L^{-1}) were determined weekly / biweekly (*n* = 320) with a bbe FluoroProbe (Moldaenke, Germany) in 1 m steps between 0 m and 120 m. Chl *a* concentrations were multiplied with the adequate lake volumes in 1 m steps, and integrated over the entire water column. Biovolumes of *P. rubescens* populations (mm^3 L^{-1}) were determined monthly (*n* = 144) and are based on microscopic counts and filament length measurements in Lugol's iodine fixed samples. Cumulative biovolumes (cm^3 m^{-2}) of *P. rubescens* for the entire water column were computed for the depth layers 0 – 20 m, 20 – 40 m and 40 – 135 m (for details see Material and Methods). (**B**) Biovolume data for three depth zones as in (**A**) but as percentages of the total biovolume.

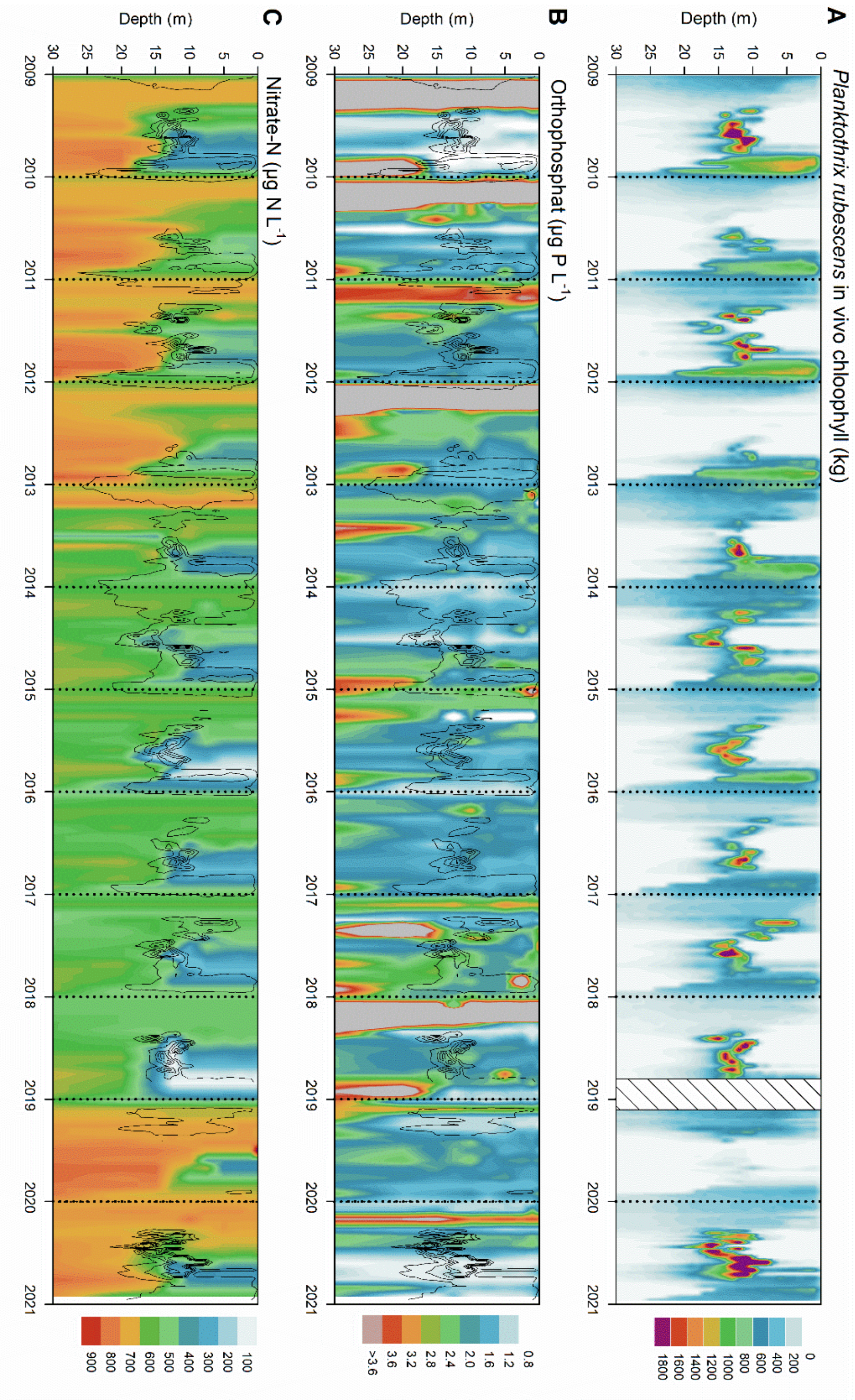

Supplementary Figure S3: Trends (2009-2020) in *Planktothrix rubescens* in vivo chlorophyll values, orthophosphate concentrations and nitrate concentrations in Lake Zurich (Switzerland) for the first 30 m of the water column. (**A**) *In vivo* chlorophyll *a* concentration (µg Chl *a* L^{-1}) of the cyanobacterium *P. rubescens* based on weekly / biweekly profiles (*n* = 320) of parameters, measured with multi-parameter probes and interpolated in 1 m steps between 0 to 30 m depth. White striped area: no measurements from November 2018 to January 2019. (**B**) Concentrations of orthophosphate (µg P L^{-1}) and (**C**) nitrate nitrogen (µg N L^{-}1) with black isoline indicating the center of the *P. rubescens* population. Panels (**B**-**C**) are based on monthly analyses (*n* = 144) of 10 sampling depths between 0 m to 30 m.
